# Preise in der Pathologie – eine Männerdomäne?

**DOI:** 10.1007/s00292-023-01239-9

**Published:** 2023-10-20

**Authors:** Elisa Malik, Thorsten Halling, Annegret Dreher, Chantal Marazia, Irene Esposito, Adrian Loerbroks, Nils Hansson

**Affiliations:** 1 Institut für Geschichte, Theorie und Ethik der Medizin, Centre for Health and Society, Medizinische Fakultät, Heinrich-Heine-Universität Düsseldorf, Moorenstr. 5, 40225 Düsseldorf, Deutschland; 2 Institut für Arbeits-, Sozial- und Umweltmedizin, Centre for Health and Society, Medizinische Fakultät, Heinrich-Heine-Universität Düsseldorf, Düsseldorf, Deutschland; 3 Institut für Pathologie, Medizinische Fakultät, Heinrich-Heine-Universität Düsseldorf, Düsseldorf, Deutschland

**Keywords:** Medaillen, Anerkennung, Auszeichnungen, Frauen in der Medizin, Gender Gap, Medals, Recognition, Awards, Women in medicine, Gender Gap

## Abstract

**Hintergrund:**

Preise verhelfen ihren Empfänger*innen zu Ruhm, Anerkennung und erleichtern in der Folge durch erhöhte Sichtbarkeit anschließende Veröffentlichungen und die Akquise von Drittmitteln. Wir stellen die zu prüfende Hypothese auf, dass Frauen trotz zunehmender Vertretung in der Pathologie als Preisträgerinnen in der Fachgesellschaft unterrepräsentiert sind und folglich ein damit verbundenes Ungleichgewicht zwischen den Geschlechtern besteht.

**Material und Methoden:**

Ausgewertet wurden veröffentlichte Daten der Deutschen Gesellschaft für Pathologie zu Preisträger*innen im Jahreszeitraum von 2000 bis 2022. Betrachtet wurden Preise, die dem Fachgebiet der Pathologie gewidmet sind. Zudem wurden die öffentlich verfügbaren Daten der Bundesärztekammer zu Geschlechter- und Altersverteilung von Pathologinnen und Pathologen in Deutschland als Referenzmaterial betrachtet.

**Ergebnisse:**

Es wurden insgesamt 6 verschiedene Preise in der Analyse berücksichtigt. Unter den 143 Preisträger*innen bei insgesamt 150 Einzelpreisvergaben im Zeitraum 2000 bis 2022 waren 55 der Preisträger*innen weiblich. Somit waren durchschnittlich 38,4 % der Preisträger*innen weiblich bei einem durchschnittlichen Pathologinnenanteil von 31 % in der deutschen Fachärzteschaft für Pathologie über den betrachteten Zeitraum von 23 Jahren. Dies bedeutet, dass die Preisträgerinnen in der Pathologie, wenn man die nationalen Statistiken zum Frauenanteil unter den Fachärzten in der Pathologie berücksichtigt, nicht unterrepräsentiert waren.

Betrachtet man allerdings die Verteilung von Preisträgerinnen in den einzelnen Preiskategorien bzw. Preisen, dann konnte beobachtet werden, dass Frauen vermehrt bei weniger prestigeträchtigen Forschungs- und Promotionspreisen vertreten waren, Männer hingegen einen großen Anteil der Preisträger*innen von Ehrenpreisen (0 % Frauenanteil) und prestigeträchtigen Preisen ausmachten (17 % Frauenanteil).

## Abstract (en)

**Background:**

Awards provide their recipients with fame and recognition, and subsequently facilitate publications and acquisition of external funding through increased visibility. We hypothesize that despite increasing representation in pathology, women are underrepresented as awardees in the German Society of Pathology and consequently there is an associated imbalance between genders.

**Material and methods:**

Published data from the German Society of Pathology on female awardees during the period from 2000 to 2022 were examined. Only awards specifically dedicated to the field of pathology were considered. In addition, the publicly available data of the German Medical Association on gender and age distribution of pathologists in Germany were considered as reference material.

**Results:**

A total of six different awards were included in the analysis. Among the 143 awardees across 150 individual awards in the period from 2000 to 2022, 55 (38.4%) of the awardees were female compared to an average percentage of 31% of women working in the field of pathology in the 23-year period under consideration. Consequently, female awardees in pathology were not underrepresented when compared to the national figures on the proportion of women in the field of pathology.

However, the distribution of female awardees across individual awards suggests that women were increasingly represented in less prestigious research and doctoral awards, while men made up a large proportion of awardees of honorary awards (0% women) and prestigious awards (17% women).

Im September 2022 veröffentlichte die Nationale Akademie der Wissenschaften Leopoldina eine Stellungnahme zu Frauen in der Wissenschaft. Diese stellt unter anderem fest, dass Frauen nach wie vor in Führungspositionen in deutschen Universitäten und Universitätskliniken deutlich unterrepräsentiert sind [1]. Die Stellungnahme erwähnt auch direkt die Vergabe von Preisen, so etwa im Kapitel „Zukunft“: „Diese Parität ist ein gesamtgesellschaftliches Ziel. Sie sollte in Institutionen wie Akademien, Hochschulen und außeruniversitären Forschungseinrichtungen ebenso wie bei der Planung von Verbundprojekten und der Vergabe von Preisen und Auszeichnungen gelten“ [1]. Ziel ist somit das Erreichen einer Geschlechterparität in wissenschaftlichen Führungspositionen, die unter anderem auch bei der Vergabe von Preisen und Auszeichnungen gelten soll. Um diese Geschlechterparität zu erreichen, schlägt die Leopoldina 4 Maßnahmen zur Veränderung dieser Unterrepräsentanz vor: 1. Strukturen ändern, 2. Frauen ermächtigen, 3. Frauen sichtbar machen und 4. Fortschritte dokumentieren und überprüfen [1]. In diesem Rahmen ist auch die Umbenennung von Zeitschriften zeitgemäß und fördernd (zum Beispiel von *Der Pathologe* in *Die Pathologie* im Jahr 2022): Sie spiegelt diese Kultur der Anerkennung wider und treibt sie voran.

Die Tendenz der Unterrepräsentanz von Frauen in den Wissenschaften zeigt sich ebenfalls im Bereich der Medizin. Frauen machen zwar den größten Anteil an Medizinstudierenden aus und promovieren häufiger, jedoch habilitieren sie sich im zeitlichen Verlauf deutlich seltener als ihre männlichen Kollegen in der Medizin – 37 % habilitierte Frauen im Vergleich zu 63 % Männern (2017–2019) bei einer Geschlechterverteilung von 65 % Frauen und 35 % Männern bei Erstimmatrikulation (2001–2003) [1].

Diese Ungleichverteilung setzt sich auch in Bereichen der Anerkennung in der Wissenschaft bei der Betrachtung von Preisen und Auszeichnungen fort [3, 8, 14]. Preise verhelfen ihren Empfänger*innen zu Ruhm und monetärem Gewinn.

Zu dem sog. Gender-Award-Gap-Phänomen gibt es bereits einige Studien, insbesondere aus den Vereinigten Staaten, die die Geschlechterverteilung von Preisträger*innen in unterschiedlichen medizinischen Gesellschaften bereits näher analysiert haben. In diesen wurden beispielsweise die Unterrepräsentation von Preisträgerinnen beim Erhalt von Preisen in den amerikanischen Gesellschaften für Neurologie, Otolaryngologie, Hämatologie/Onkologie und auch in der Pathologie beschrieben – jeweils in Bezugnahme zur Geschlechterverteilung im gesamten Fachgebiet, erhoben durch die Association of American Medical Colleges (AAMC) [4, 6, 15–19]. Ebenso wurde bereits ein möglicher Gender Award Gap in der Rheumatologie in Nordamerika und Europa genauer untersucht [7]. In der Studie zur Preiskultur in der Pathologie wurde die Geschlechterverteilung bei Anerkennungspreisen der US-amerikanischen Gesellschaften für Pathologie näher analysiert und eine Unterrepräsentation von Frauen festgestellt [18]. Eine Untersuchung der Geschlechterverteilung zu Preisempfänger*innen in der Pathologie in Deutschland steht unseres Wissens noch aus und diese Forschungslücke soll im Rahmen der vorliegenden Arbeit geschlossen werden.

Der weibliche Anteil unter Pathologinnen in der deutschen Ärzteschaft betrug in den Jahren 2000–2022 im Durchschnitt 31 % [9]. Allerdings nimmt der Anteil an Frauen im Fachgebiet der Pathologie stetig zu. So ist der Anteil weiblicher und in Deutschland tätiger Patholog*innen unter 40 zwischen 2000 und 2022 um 20 % gestiegen [9]. Es stellt sich die Frage, ob die zunehmende „Feminisierung“ der Pathologie mit der Anerkennung und der Preiskultur des Faches einhergeht. Mit dieser Arbeit möchten wir einen möglichen Gender Award Gap in der Pathologie in Deutschland betrachten und analysieren hierzu im Zeitraum 2000–2022 Auszeichnungen, die im Fachgebiet der Pathologie vergeben wurden.

## Material und Methoden

Mithilfe veröffentlichter Daten zu Preisträger*innen von Preisen im Fachbereich der Pathologie, verliehen durch die Deutsche Gesellschaft für Pathologie (DGP), analysierten wir die Geschlechterverteilung der Preisempfänger*innen im Fachbereich der Pathologie (für alle Preise und pro Preis, s. Abschnitt Ergebnisse) und setzten diese Daten in Bezug zum Anteil an Pathologinnen in der deutschen Ärzteschaft. Die Daten entstammen der Internetseite der DGP sowie veröffentlichten Schriften zu Jahrestagungen der DGP [13].

Preisträger*innen müssen nicht zwingend Mitglied der Fachgesellschaft sein, weshalb in dieser Arbeit öffentlich verfügbare Daten der Bundesärztekammer zur Geschlechter- und Altersverteilung der Ärzteschaft in der Pathologie über den Zeitraum 2000–2022 als Referenz herangezogen wurden [9].

Die DGP umfasst 1071 Mitglieder (m 749/w 322, Stand Dezember 2022), darunter 84 Juniormitglieder (Assistenzärzt*innen bis 6 Jahre nach der Approbation; Naturwissenschaftler*innen bis zu 6 Jahre nach dem jeweiligen Studienabschluss) (m 45/w 39) und 188 Ruheständler*innen (m 176/w 12). Die DGP verleiht in unterschiedlichen Zeitintervallen jeweils insgesamt 6 Preise: den Rudolf-Virchow-Preis, die Rudolf-Virchow-Medaille, den Posterpreis, den Promotionspreis, den DGP-Forschungspreis und gemeinsam mit Novartis Oncology den Novartis-Preis [11, 12].

Bei der Auswahl der Preise wurden die spezifisch auf den Fachbereich Pathologie bezogenen Preise berücksichtigt. Der Hochgesand-Preis als Auszeichnung im Fachgebiet der Pathologie wurde im Jahre 2017 erstmals eingeführt und bislang zweimal (2017 und 2021) durch die Hochgesand-Stiftung verliehen [10]. Aufgrund der geringen Preisanzahl im Untersuchungszeitraum wurde deshalb auf den Einschluss in die Untersuchung verzichtet. Preise, die einen größeren Themenbereich wie die Krebsforschung (z. B. der Gerhard-Domagk-Preis oder der Hella-Bühler Preis) und somit andere Fachgebiete wie die Hämatologie, Urologie, Gynäkologie umfassen, wurden nicht einbezogen.

Das Geschlecht der jeweiligen Preisträger*innen wurde anhand des Vornamens festgestellt. War der Vorname nicht angegeben, wurde mithilfe einer Onlinerecherche der korrekte Vorname ermittelt. Eine Person wurde aufgrund des nicht ermittelbaren Vornamens aus der Datensammlung ausgeschlossen. Zusätzlich wurde die Zusammensetzung der Jury des Rudolf-Virchow-Preises nach Geschlecht näher betrachtet.

In dieser Analyse haben wir die Preise in der Pathologie in 2 Kategorien eingeteilt:

Ehrenpreise, die Persönlichkeiten im Fachgebiet der Pathologie und ihre Verdienste ehren, und Forschungspreise, die meist eine herausragende wissenschaftliche Arbeit auszeichnen. Dazu gehören auch Poster- und Promotionspreise:

1. Ehrenpreise
  Rudolf-Virchow-Medaille
2. Forschungspreise
  Rudolf-Virchow-Preis
  DGP-Forschungspreis
  Posterpreis
  Promotionspreis
  Novartis-Preis

Die DGP hebt 2 Auszeichnungen besonders hervor: die Rudolf-Virchow-Medaille und den Rudolf-Virchow-Preis. Beide Preise können somit als prestigeträchtig betrachtet werden [11].

## Ergebnis

**Figure 1:**
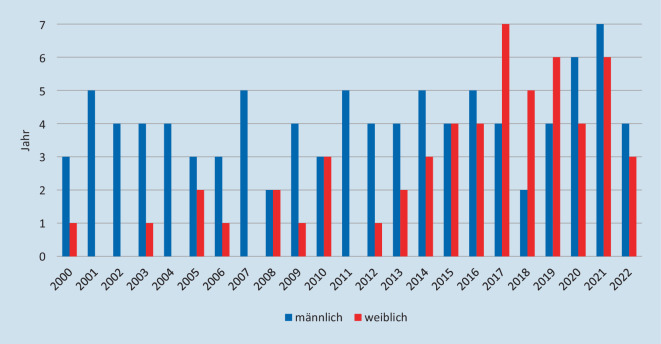

Die Deutsche Gesellschaft für Pathologie vergibt insgesamt 6 Auszeichnungen für Erkenntnisse und Fortschritte spezifisch für die Pathologie (Abb. 1).

**Figure 2:**
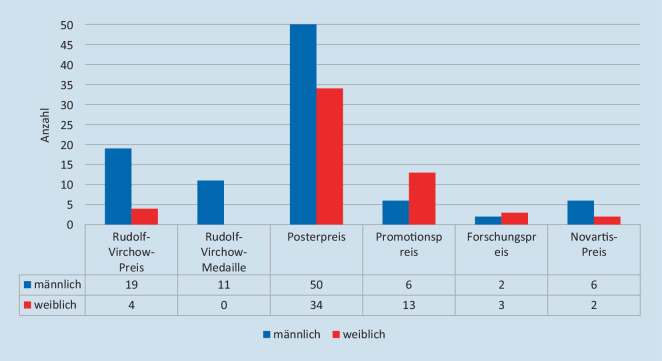

Im Zeitraum von 2000 bis 2022 konnten wir bei 150 Einzelpreisverleihungen insgesamt 143 Preisträger*innen identifizieren, die für ihre Forschung und Arbeit in der Pathologie ausgezeichnet wurden (Abb. 2). Die meisten Empfänger*innen erhielten nur eine Auszeichnung (*n* = 137). Insgesamt 5 Preisträger*innen erhielten während des Untersuchungszeitraums 2 Auszeichnungen und ein Preisträger erhielt insgesamt 3 Auszeichnungen.

**Figure 3:**
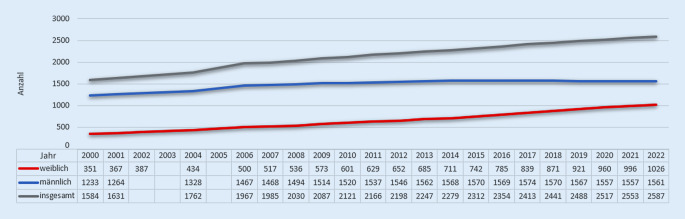

Die geschlechterspezifische Verteilung aller Auszeichnungen zeigt, dass Frauen als Preisträger*innen in diesem Zeitraum nicht unterrepräsentiert sind. Bei insgesamt 143 ausgezeichneten Mediziner*innen und 150 Einzelpreisen lag die Anzahl der Frauen bei 55 (38,4 %) mit insgesamt 56 Auszeichnungen bei einem durchschnittlichen Frauenanteil von 31 % in der Pathologie (Tab. 1). Eine Teilung dieses Beobachtungszeitraumes in 3 Zeitabschnitte zeigt eine deutliche durchschnittliche Zunahme des Anteils an Preisen, die an Frauen vergeben wurden. So betrug der Frauenanteil in der Periode 2000–2007 13,89 %, 2008–2015 34,78 % und 2016–2022 52,2 %. In diesem Zeitfenster nimmt auch der Anteil von Frauen unter Patholog*innen zu. Zwischen 2000 und 2007 betrug der Frauenanteil 28,6 %, zwischen 2008 und 2015 29,4 % und zwischen 2016 und 2022 42,8 % ([9]; Abb. 3).

**Table 1:** 

Jahr		Rudolf-Virchow-Preis	Rudolf-Virchow-Medaille	Posterpreis	Promotionspreis	Forschungspreis	Novartis-Preis	Preise gesamt	Anteil weiblich in %
*2000*	M	1	–	2	–	–	–	3	–
	W	–	–	1	–	–	–	1	25
*2001*	M	1	1	3	–	–	–	5	–
	W	–	–	–	–	–	–	0	0
*2002*	M	1	–	3	–	–	–	4	–
	W	–	–	–	–	–	–	0	0
*2003*	M	1	1	2	–	–	–	4	–
	W	–	–	1	–	–	–	1	20
*2004*	M	1	–	3	–	–	–	4	–
	W	–	–	–	–	–	–	0	0
*2005*	M	1	1	1	–	–	–	3	–
	W	–	–	2	–	–	–	2	40
*2006*	M	1	–	2	–	–	–	3	–
	W	–	–	1	–	–	–	1	25
*2007*	M	1	1	3	–	–	–	5	–
	W	–	–	–	–	–	–	0	0
*2008*	M	1	–	1	–	–	–	2	–
	W	–	–	2	–	–	–	2	50
*2009*	M	1	1	2	–	–	–	4	–
	W	–	–	1	–	–	–	1	20
*2010*	M	1	–	2	–	–	–	3	–
	W	–	–	3	–	–	–	3	50
*2011*	M	1	1	3	–	–	–	5	–
	W	–	–	–	–	–	–	0	0
*2012*	M	–	–	3	–	–	1	4	–
	W	1	–	–	–	–	–	1	20
*2013*	M	1	1	1	1	–	–	4	–
	W	–	–	2	–	–	–	2	33
*2014*	M	1	–	2	2	–	–	5	–
	W	–	–	1	1	–	1	3	38
*2015*	M	–	1	3	–	–	–	4	–
	W	1	–	1	2	–	–	4	50
*2016*	M	1	–	2	–	–	2	5	–
	W	–	–	2	2	–	–	4	44
*2017*	M	–	1	–	1	2	–	4	–
	W	1	–	4	1	1	–	7	64
*2018*	M	1	–	1	–	–	–	2	–
	W	–	–	2	2	–	1	5	71
*2019*	M	–	1	2	1	–	–	4	–
	W	1	–	2	2	1	–	6	60
*2020*	M	1	–	3	–	–	2	6	–
	W	–	–	3	1	–	–	4	40
*2021*	M	1	1	4	1	–	–	7	–
	W	–	–	4	1	1	–	6	46
*2022*	M	1	–	2	–	–	1	4	–
	W	–	–	2	1	–	–	3	43
*Summe* *2000 bis 2022*	M	19	11	50	6	2	6	94	–
	W	4	0	34	13	3	2	56	37
	% W	17 %	0 %	41 %	68 %	60 %	25 %	37 %	–
	Total	23	11	84	19	5	8	150	–

In dieser Analyse teilten wir die Preise in 2 Kategorien ein, und zwar in Ehrenpreise und in Forschungspreise.

Der exakte Fisher-Test wurde angewandt, um festzustellen, ob ein signifikanter Zusammenhang zwischen Geschlecht und Art des vergebenen Preises (Ehrenpreis vs. Forschungspreis) besteht. Es wurde ein statistisch signifikanter Zusammenhang zwischen beiden Variablen festgestellt (zweiseitiger Test *p* = 0,007).

Die Rudolf-Virchow-Medaille, die Personen auszeichnet, die sich „um die Entwicklung der Pathologie besonders verdient gemacht haben“ [11] und der der einzige Ehrenpreis in unserer Analyse ist, wurde bis zum jetzigen Zeitpunkt mit 11 Preisverleihungen im betrachteten Zeitraum von 23 Jahren noch nie an eine Frau verliehen. Diesen Ehrenpreis haben in den letzten 23 Jahren 11 Männer zwischen 66 und 83 Jahren erhalten.

Insgesamt 5 Preise zeichnen verschiedene Forschungsarbeiten in Form von Forschungs‑, Promotions- und Posterpreisen aus. Diese wurden in dieser Arbeit unter der Kategorie „Forschungspreise“ zusammengefasst. Von 132 ermittelten Preisträger*innen erhielten insgesamt 55 Frauen (41,67 %) 56 Forschungspreise (eine Preisträgerin mit zweifacher Auszeichnung) bei einem Frauenanteil von 31 % unter Patholog*innen.

Beim Promotionspreis sowie beim DGP-Forschungspreis erhielten Frauen mit 68,42 % bzw. 60 % die jeweilige Auszeichnung und waren somit überrepräsentiert. Bei dem prestigeträchtigen Rudolf-Virchow-Preis sind Frauen allerdings unterrepräsentiert (17,39 % Frauenanteil). Dies gilt auch für den Novartis-Preis (25 % Frauenanteil). Der Posterpreis zeigte mit 40,5 % eine annähernd ausgeglichene Verteilung an weiblichen und männlichen Preisträger*innen über die letzten Jahre.

Der Anteil an Pathologinnen unter 40 – eine Altersgrenze, die bei dem prestigeträchtigen Rudolf-Virchow-Preis ein Einschlusskriterium darstellt – betrug zwischen 2006 und 2022 durchschnittlich 51 %. Zwischen 2006 und 2010 betrug der Frauenanteil unter jungen Patholog*innen durchschnittlich 42,4 %, zwischen 2011 und 2016 55,33 % und in den letzten Jahren zwischen 2017 und 2022 59 % (Tab. 2). Diese Daten weisen auf eine langsam aber stetig wachsende Zahl an jungen Pathologinnen hin [9]. Eine spezifische Altersgrenze gibt es bei den anderen Forschungspreisen nicht, jedoch richten sich diese vermehrt an junge Wissenschaftler*innen.

**Table 2:** 

Jahr	Unter 40 J. insg.	Unter 40 J. weiblich	Weiblicher Anteil bis 40 J. (in %)
2006	149	59	40
2007	134	53	40
2008	126	46	37
2009	142	63	44
2010	166	84	51
2011	175	91	52
2012	164	88	54
2013	191	106	55
2014	188	106	56
2015	204	119	58
2016	211	120	57
2017	216	122	56
2018	225	123	55
2019	236	145	61
2020	238	146	61
2021	238	143	60
2022	244	149	61
*Ergebnis*			*53*

Unter diesem Aspekt der Altersstruktur kann man die Preisverteilung des Rudolf-Virchow-Preises mit Einschlusskriterium „Alter unter 40 Jahre“ näher begutachten. Vergleicht man die Anzahl an Preisträgerinnen und den Anteil an Pathologinnen unter 40 Jahren in der deutschen Ärzteschaft, so lässt sich eine deutliche Unterrepräsentation feststellen. Bei einem durchschnittlichen Anteil an Pathologinnen unter 40 Jahren von 42 % zwischen 2006 und 2010 gab es in diesem Zeitraum keine Preisträgerinnen. Zwischen 2011 und 2016 betrug der Preisträgerinnenanteil 33 % bei einem Anteil junger Pathologinnen von 55 % in der Ärzteschaft der Pathologie unter 40 Jahren und zwischen 2017 und 2022 waren unter den Preisträger*innen 33 % weiblich bei einem Frauenanteil von 59 % in der Ärzteschaft der Pathologie unter 40 Jahren.

## Diskussion

Dies ist unseres Wissens nach die erste Studie, die sich mit der Geschlechterrepräsentation von Preisen im Fachgebiet der Pathologie in Deutschland auseinandersetzt. Insgesamt konnten wir zeigen, dass Frauen als Preisträgerinnen in der Pathologie nicht unterrepräsentiert sind. Bei einem durchschnittlichen Frauenanteil von 31 % in der deutschen pathologischen Ärzteschaft wurden 37,3 % der Preise an Frauen verliehen. Im Untersuchungszeitraum zeigte sich eine deutliche durchschnittliche Zunahme des Anteils an Preisen, die an Frauen vergeben wurden, bei ebenfalls einer Zunahme des Frauenanteils unter Patholog*innen [9].

Eine aktuelle Studie aus den Vereinigten Staaten zur Geschlechterverteilung von Preisen in der Pathologie hingegen stellte, wie bereits erwähnt, fest, dass Frauen bei Anerkennungspreisen der US-amerikanischen Fachgesellschaften für Pathologie unterrepräsentiert sind. In dieser Studie wurde zudem implizit auf eine deutliche Unterrepräsentation von Frauen bei prestigeträchtigen Preisen hingewiesen [18]. Vergleichbare Ergebnisse zu den unsrigen hinsichtlich einer ausgeglichenen Geschlechterverteilung bei Preisträger*innen ergaben sich in einer anderen Studie zu Preisen in der Chirurgie auch in den Vereinigten Staaten [2]. Hier wurde zudem ebenfalls auf eine unverhältnismäßige geringe weibliche Vertretung unter prestigeträchtigen Preisen hingewiesen. Ähnliches konnte für die Verteilung von Frauen unter den prestigeträchtigen Preisen in der Pathologie nun im Rahmen der vorliegenden Studie erstmals auch in Deutschland festgestellt werden.

Einer der prestigeträchtigsten Ehrenpreise in Deutschland ist die Rudolf-Virchow-Medaille. Der jeweilige Preisträger oder die jeweilige Preisträgerin muss bereits ein hochgeachtetes Lebenswerk geschaffen haben, um sich „um die Entwicklung der Pathologie besonders verdient“ [11] gemacht haben zu können. Hier ist ein Kohorteneffekt wahrscheinlich: Alle Personen, die bislang diesen Preis erhalten haben, sind männlich und älter als 66 Jahre. Etwa 10 % der berufstätigen Patholog*innen über 65 Jahre waren in diesem Zeitraum Frauen. Dieser Wert spiegelt nicht die tatsächliche Zahl der Pathologinnen über 65 wider (beispielsweise emeritierte), sondern lediglich die der berufstätigen Pathologinnen über 65. Empfänger*innen der Medaille sind Ordinarien, die zum Zeitpunkt der Verleihung nicht mehr berufstätig waren. Jedoch kann man durch diese Zahlenwerte durchaus auf einen verminderten weiblichen Anteil in der Ärzteschaft der Pathologie im Alter von über 65 Jahren schließen. Diese Zahlen geben aber nur einen Hinweis auf einen möglichen Zustand; tatsächliche Daten hierzu waren online nicht verfügbar. Die Anzahl an berufstätigen Pathologinnen über 65 Jahre stieg von 7 % (2006) auf 11 % (2012) auf 16 % (2018) auf 19 % (2022) und zeigt auch nochmals, dass der weibliche Anteil unter Patholog*innen über die Zeit gewachsen ist [9]. Man könnte zumindest annehmen, dass diese Zahlenwerte und die Zunahme des weiblichen Anteils in der Pathologie in ähnlicher Weise auch auf nicht mehr berufstätige, emeritierte Patholog*innen übertragbar sind.

Obgleich der Kohorteneffekt das Fehlen von weiblichen Preisträgern der Rudolf-Virchow-Medaille erklären könnte, lässt sich dieser Gedankengang nicht auf den prestigeträchtigen Rudolf-Virchow-Preis übertragen. Dieser wird an junge Patholog*innen „unter 40 Jahren für eine noch nicht veröffentlichte oder eine nicht länger als ein Jahr vor der Bewerbung publizierte wissenschaftliche Arbeit“ [11] verliehen und wurde in den letzten 23 Jahren an 4 Frauen bei insgesamt 23 Preisträger*innen verliehen (17 % Frauenanteil). Alle Preisträger*innen in den Jahren 2000–2011 waren Männer. Ab 2012 betrug der Frauenanteil 36 %, was nicht den durchschnittlichen Frauenanteil unter Patholog*innen unter 40 Jahren (57 % Frauenanteil) widerspiegelt.

In den letzten Jahren ist der Anteil an Preisträgerinnen deutlich gestiegen parallel mit der Zusammensetzung der Auswahlgremien, in denen Frauen inzwischen zwar immer noch eine geringere, aber stärkere Vertretung aufweisen. Beobachtet man die Zusammenstellung der Jury des Rudolf-Virchow-Preises, so ist zu erkennen, dass ab 2010 immer eine Frau bei 6–7 Jurymitgliedern und ab 2015 immer 2 Frauen bei 5–6 Jurymitgliedern vertreten sind. Im Zeitraum 2010–2015, in dem jährlich eine Frau in der Jury vertreten war, erhielt die allererste Frau die Auszeichnung, ab 2015 – mit stets 2 weiblichen Jurymitgliedern – erhielten 3 Frauen die prestigeträchtige Auszeichnung [13].

Eine weibliche Vertretung ist ebenfalls im Vorstand der Deutschen Gesellschaft für Pathologie vorzufinden, welche über die Vergabe der prestigeträchtigen Rudolf-Virchow-Medaille entscheidet. Ein Kausalitätszusammenhang zwischen geschlechterspezifischer Verteilung in den Gremien und Vergabe von Preisen lässt sich hieraus nicht zwingend ableiten. Die Zunahme der weiblichen Vertretung sowohl unter den Preisträger*innen als auch im Fachgebiet der Pathologie deutet auf eine weitere Zunahme von Frauen auch in Auswahlgremien hin.

Die Metapher der „leaky pipeline“ beschreibt das Ausscheiden von Frauen aus der Wissenschaft im zeitlichen Verlauf sowie im Karriereverlauf. Ein in diesem Rahmen genanntes Argument ist, dass Frauen noch nicht lange genug im jeweiligen Wissenschaftsgebiet tätig waren, um eine Führungsposition zu erhalten [5]. Das Argument der fehlenden, qualifizierten Frauen in der „leaky pipeline“, welches ebenfalls von anderen Arbeiten zur Geschlechterverteilung aufgegriffen wird [2, 18], erklärt jedoch nicht das Ergebnis und stellt als alleiniger Grund keine Ursache für die Geschlechterunterschiede dar. Dieses Argument wurde bereits 2008 in der Arbeit von Molly Carnes et al. widerlegt [5]. Der Frauenanteil und damit auch die Anzahl an qualifizierten Pathologinnen der Pipeline ist in den letzten Jahren deutlich gestiegen. Mit steigendem Frauenanteil in der Pathologie gewannen zwar in den vergangenen Jahren mehr Frauen Preise, jedoch waren sie bei Prestigepreisen immer noch deutlich unterrepräsentiert.

Unsere Analyse weist Limitationen in Bezug auf die Methode der Geschlechteridentifizierung auf, da sie allein auf einer Vornamenzuordnung beruht.

## Schlussfolgerung

Zwischen 2000 und 2022 waren Pathologinnen unter den Preisträger*innen im Durchschnitt aller betrachteten Preisen nicht unterrepräsentiert. Betrachtet man jedoch diesen Zeitabschnitt differenzierter, so fällt auf, dass Frauen in den Jahren 2000 bis 2007 unterrepräsentiert waren bei einem durchschnittlichen Frauenanteil von 28,6 % in diesem Zeitraum. In den Folgejahren stieg der Frauenanteil bei Preisen von 34,78 % (2008–2015) auf 50,7 % (2015–2022) bei einem durchschnittlichen Frauenanteil unter Patholog*innen von 29,4 und 42,8 % in denselben Zeitabschnitten. Allerdings sind Frauen bei Prestigepreisen immer noch sehr stark unterrepräsentiert.

Zur Analyse möglicher Gründe und Kausalitäten des Gender Award Gaps sind weitere Studien auf einer breiteren Quellengrundlage notwendig. Dazu gehört beispielsweise die Frage der Geschlechterverteilung bei der Besetzung von Preisgremien, bei der Bearbeitung bestimmter Themenfelder oder des Einflusses der persönlichen Lebensgestaltung in Beruf und Familie auf die jeweilige Sichtbarkeit in der Scientific Community und damit auch auf die Chancen, Forschungspreise zu erhalten.
